# Changing geographies of fashion during COVID‐19: The Australian case

**DOI:** 10.1111/1745-5871.12460

**Published:** 2021-03-04

**Authors:** Taylor Brydges, Lisa Heinze, Monique Retamal

**Affiliations:** ^1^ Department of Human Geography Stockholm University Stockholm Sweden; ^2^ Institute for Sustainable Futures University of Technology Sydney Ultimo New South Wales Australia; ^3^ Sydney Environment Institute University of Sydney Sydney New South Wales Australia

**Keywords:** Australia, COVID‐19, fashion, local manufacturing, path dependency, spaces of design

## Abstract

COVID‐19 has impacted a range of industries, of which the fashion industry is no exception. Here, we examine the effects of COVID‐19 on the geographies of that industry in Australia. We use a path‐dependency framework to understand the evolution of the Australian fashion industry and to evaluate the impact of COVID‐19 as an external shock to this evolution. The Australian fashion industry has been hit particularly hard by the pandemic. Drawing upon a qualitative methodology and associated set of methods consisting of 24 semi‐structured interviews with key industry actors, we explore three key themes. First, we examine the impact of COVID‐19 on firm dynamics, investigating how fashion brands have navigated a period of significant uncertainty through a combination of highly nimble internal business practices and industry collaborations. Second, in light of unstable globalised supply chains, we consider the important role of local garment production in supporting industrial resilience. Third, we explore how changing consumption behaviours are altering the retail geographies of the Australian fashion industry in both physical and virtual spaces. Based on our analysis of preliminary results, we reflect on the suitability of an evolutionary approach in this context and outline a number of themes of future research.

**Key insights**

Taking a path‐dependency approach, we demonstrate how the ongoing impact of COVID‐19 is reshaping the geographies of the Australia fashion industry across the spaces of design, production, and retail. In the midst of crisis, those in the Australian fashion industry have been able to build upon several existing strengths, including a burgeoning design reputation and local garment manufacturing, while also developing new industrial pathways to harnessing social media and digital technologies. Challenges facing the sector are identified, among them the need for investment in local manufacturing capabilities, and themes for future research are shared.

## 1 INTRODUCTION

Like so many sectors of the economy, the fashion industry has been significantly impacted by the COVID‐19 pandemic. Before the onset of COVID, the industry was already undergoing several transformations brought on by factors including digitalization and sustainability that were challenging dominant industry practices and spatialities (Crewe, 2017). In this article, we report on our investigation of the ongoing impact of COVID‐19 on the geographies of the Australian fashion industry. To do so, we put COVID‐19 in context by using a path‐dependency framework to understand the evolution of the Australian fashion industry and to evaluate the pandemic's impact as a shock external to this evolution. The concept of path dependency allows for an understanding of the key dynamics that led to the formation and structure of the contemporary Australian fashion industry and, in turn, permits us to consider how the industry is experiencing and responding to the challenges of COVID‐19. From our preliminary analysis of empirical material, we highlight three key spaces where the geographies of Australian fashion have been significantly impacted thus far: firm‐level dynamics, garment production, and retail.

Australia has been chosen as a case study location for a number of reasons. A tier‐two fashion country, Australia has long been home to a vibrant domestic fashion industry that, in recent years, has experienced an increase in popularity abroad^1^ (English & Pomazan, 2010). Yet according to an April 2020 survey by the Australian Fashion Council (AFC) and McKinsey & Company, two‐thirds of industry members reported that they will not be able to financially recover from the pandemic (AFC, 2020a, 2020b). Indeed, even before the pandemic, the Australian fashion industry was not immune to challenges brought on by factors including the growth of online retailers, changing consumer preferences, and competition from fast fashion retailers such as Zara, H&M, and Uniqlo (BBC, 2019).

Empirically, we draw upon a qualitative methodology and associated set of methods consisting of semi‐structured interviews conducted between June and October 2020 with 24 industry key informants (including fashion designers, representatives from industry associations, consumers, and retailers; see Appendix A). Interviews were transcribed and coded to theme (Crang, 2005; James, 2006) and supplemented by a review of media articles and industry reports on the impact of COVID‐19 on the Australian fashion industry between mid‐March 2020 and early‐August 2020. That work forms part of a broader project examining sustainability and industrial dynamics of the Australian fashion industry.

In exploring how COVID‐19 is (re)shaping the geographies of the Australian fashion industry, we aim to make three contributions to the knowledge base. First, we contribute to the literature on path dependency by offering a case of an understudied industry as well as by exploring an industrial response to a dramatic external shock. Second, we contribute to the theorization of the contemporary Australian fashion industry. Third, we offer an early empirical account of the impact of COVID‐19 on industrial dynamics.

## 2 PUTTING COVID‐19 IN CONTEXT: A PATH‐DEPENDENCY APPROACH

From the subdiscipline of economic geography, the concept of path dependency is used to examine the historical evolution of regions over time (cf. Boschma & Lambooy, 1999; Boschma & Martin, 2010; Cooke & Morgan, 1998; Martin & Sunley, 2006; Maskell & Malmberg, 1999; Storper, 1995). Path dependency helps us understand the historical processes underpinning the development of regions, industries, and/or technologies, accounting for the place‐dependent nature of these transformations (Boschma & Martin, 2010; Hodgson, 2001).

From an evolutionary perspective, specialisation can become reinforced by various actions or initiatives such as policy interventions, that lead over time to the build‐up of expert knowledge and capabilities in a particular region (cf. Boschma & Martin, 2010; Coenen et al., 2017; Couch et al., 2011). Path formation is thus highly contextualised, uneven, and place based (Boschma & Martin, 2010; Couch et al., 2011; Gertler, 2005; Schienstock, 2007). However, it can also lead to industrial “lock in” (Arthur, 1989) whereby regional specialisation is reproduced and reinforced to a point where it can become difficult to ignite new forms of specialisation (Martin, 2010; Martin & Sunley, 2006).

A strength of path‐dependency theory is it emphasises the importance of historical structural factors that play critical roles in shaping the practices and relations of firms and/or industries (Rantisi, 2004, p. 88). However, paths are not necessarily linear or fixed (Hassink, 2005; Martin, 2010; Strambach, 2008). As Rantisi (2004, p. 104) writes, “historical ruptures or crises unleash opportunities for altering a region‐industrial trajectory.” New paths can be created over time, resulting most commonly from exogenous shocks, internal disturbances, or a combination of the two (Hassink, 2005; Rantisi, 2004; Schienstock, 2007; Strambach & Klement, 2013). This dynamic interplay is a key reason why a path‐dependency framework is appropriate for examining the impact of COVID‐19 on the evolution of the Australian fashion industry.

Moreover, although structural factors play an important role in these processes, it is also important to recognise the agency of individual actors acting and responding to unique historical and political contexts (Rantisi, 2004). For example, Rantisi (2002, 2004) provides an illustrative case study of path‐dependent dynamics at work in the New York fashion industry. In that case, the combination of historical advantages tied to manufacturing capabilities and the presence of innovative trade groups and actors who adapted existing institutional infrastructures to chart new paths ultimately led to the transition of the city's fashion industry from a hub for garment manufacturing to the locus of a glamorous fashion capital.

In the next section, we take a path‐dependent approach to trace the evolution of the Australian post‐war fashion industry in which we have identified three key phases.

## 3 PATH DEPENDENCY IN THE AUSTRALIAN FASHION INDUSTRY

### 3.1 Phase 1: Importing international style

Beginning in the post‐war period,^2^ Australian fashion was defined by increasing levels of consumerism and moves to import styles from international fashion capitals (Craik, 2015, 2017; English, 2010. As Mitchell (2010) points out, first there was the impact of Parisienne couture, which was seen as the epitome of style, catering to a clientele base of urban socialites. Fashion buyers would go to Paris to view the latest couture collections and bring back garment models (or patterns) which would be manufactured by highly skilled seamstresses in Australia.

The influence of British fashions was bolstered by a visit to Australia by Queen Elizabeth II in 1954 (Mitchell, 2010). These styles were more casual than evident in the French aesthetic and arguably were better suited to the more laid‐back Australian lifestyle and climate. Style shifted once again when American sportswear brands were imported with increasing frequency, offering an even more casual aesthetic that resonated with Australian consumers.

The importing of style had several implications for the domestic fashion industry, and Australians developed a highly and technically skilled garment manufacturing industry in cities such as Melbourne and Sydney (Mitchell, 2010). This development helped lay the foundations for the emergence of early, including haute couture, Australian fashion designers such as Beril Jents, who had the technical skills to produce high quality garments attuned to an Australian consumer aesthetic (Pomazan, 2010a).

### 3.2 Phase 2: Creating an Australian fashion identity

Challenging the tradition of importing international brands, the second key stage in the evolution of the Australian fashion industry from the 1960s onwards was the emergence of a new generation of Australian independent fashion designers looking to create their own design identities (Craik, 2017; Williamson, 2010). The use of prints and Indigenous designs became popular in the 1970s and 1980s, alongside boldly printed textiles, often incorporating local flora and fauna, and that shift in approach played an important part in the creation of a distinctly Australian fashion design aesthetic (Craik, 2009; Williamson, 2010).

In this vein, independent fashion designers such as Carla Zampatti in the 1960s, Jenny Kee^3^ and Linda Jackson in the 1970s, and Easton Pearson in the late 1980s and 1990s, among others, found mainstream commercial success and played important roles in putting the Australian fashion industry on the map (Craik, 2015; Leong & Somerville, 2010; Williamson, 2010). The articulation of this new aesthetic was supported by the growth of key industry actors, such as fashion magazine editors and photographers, who produced for an eager local audience of print fashion media consumers images of Australian fashion designs against iconic backdrops (Palmer & Rhodes, 2010).

The Australian wool and textile industries also played vital roles in supporting the evolution of the domestic fashion industry (Williamson, 2010). Research and development investment, as well as that by industry associations such as the Australian Wool Board, fuelled innovation in the sector, leading to the introduction of both new synthetics and higher quality wool production (Williamson, 2010). State investment in such initiatives, as well as engagement from new organisations such as the Fashion Design Council, also reflected a belief that fashion design and textile production played an important role in nation building and national identity (Leong & Somerville, 2010; Palmer & Rhodes, 2010; Williamson, 2010).

In summary, the mid‐century saw the formation of an emerging Australian fashion industry that began to resonate with an increasingly international audience.

### 3.3 Phase 3: Australian fashion goes global

Building on the confluence of strengths in fashion design and manufacturing, the third key phase in Australia's fashion industry evolution was its integration into the global fashion system from the 1990s onwards (Craik, 2015; Whitfield, 2010). This integration occurred even though the domestic fashion industry was reeling from a reduction of tariffs on foreign garments, which flooded the market with lower cost imports and led to mass layoffs in the textile, apparel, and footwear manufacturing sectors and to growing technological and infrastructure deficiencies in the domestic garment manufacturing industry (Craik, 2015; Kellock, 2010; Whitfield, 2010). It is estimated that employment in the textile, clothing, and footwear manufacturing sector fell from 115,700 workers in 1989 to 40,000 in 2013 (Craik, 2015). The retail landscape faced significant challenges in the early 2010s, when international fast fashion brands such as Zara, H&M, and Uniqlo entered the market, attracting customers with low prices and trendy styles (BBC, 2019).

With domestic brands and retailers facing some of their worst years on record,^4^ industry players increasingly began to look outward. One example of this response was the introduction of Australian Fashion Week as a way to showcase domestic brands to press and buyers from Australia and beyond (Weller, 2008; Whitfield, 2010). Capitalising on the opportunity to showcase and promote themselves to influential industry actors, the leads at emerging fashion brands such as Zimmerman, Colette Dinnigan, Sass & Bide, and Willow began gaining traction domestically as well as in major fashion cities such as Paris and New York (Whitfield, 2010). Their success also exemplifies how members of the Australian fashion industry came together in a collaborative manner in response to external shocks in ways that strengthened the industry. The Australian fashion industry also developed a reputation for designer womenswear and swimwear (Craik, 2009; Schmidt, 2010), both of which have found success with consumers abroad, particularly in markets in the United States and the United Kingdom^5^ (Stilinovic, 2018).

Australia is also home to a vibrant independent fashion design scene consisting of a range of business models, including small‐scale independents, commercial independents, indie fashion businesses, niche specialists, and second‐hand clothing retailers (see Tuite, 2019a). Many participants in the industry compete on quality and design rather than on cost, and many often have at their core broader sustainability and/or social impact messages related to local, sustainable or ethical production, or smart fabrics (Pomazan, 2010b; Tuite, 2019a). Several brands, including a growing number of domestic fast fashion brands (Payne, 2016) “walk the line” between being mainstream and independent to considerable success both domestically and abroad (Tuite, 2019a).

Thus, over the last 70 challenging years, members of the Australian fashion industry have created commercially viable identities initially built upon historical strengths and investments in domestic manufacturing that have increasingly found success both at home and abroad.

## 4 EMPIRICS

In this section, informed by our qualitative research and ethics‐cleared deidentified interviews with 24 members of the Australian fashion industry, we explore how the pandemic is changing the geographies of Australian fashion [ethics clearance details]. Here, we flag three key spaces that emerged from interviews—firm‐level dynamics, production, and retail—as key spaces where the path‐dependent evolution of the Australian fashion industry is being impacted by the external shock of COVID‐19.

### 4.1 Impact on firm‐level dynamics

In early April 2020, the AFC conducted a survey of the industry with McKinsey & Company in order to gauge the immediate impact of COVID‐19 on the industry.^6^ The reported impact of the pandemic was staggering: an 87% decline in in‐store sales and a 56% decline in online sales over the previous weeks (AFC, 2020a). Seemingly overnight, the industry faced a financial crisis. With stores closed and inventory mounting, a growing number of houses and associated industries faced the prospect of being unable to meet financial obligations such as bank loan repayments (51%), leases for retail (76%), and office space (60%) and wages for staff both permanent (59%) and freelance (64%) (AFC, 2020a). With 80% of apparel and fashion brand respondents agreeing or strongly agreeing with the statement they were “negatively implicated [sic] by COVID‐19,” only 34% of businesses agreed with the statement that they were “confident” in their businesses' capacities to rebound financially (AFC, 2020a). Thus, it is clear that COVID‐19 has been a significant external shock to the Australian fashion industry.

As the results of the AFC (2020a) survey suggest, COVID‐19 led the overwhelming majority of Australian fashion and apparel brands to enter survival mode in order to protect their businesses and employees. Yet our participants reported being highly nimble and responsive to industry changes. Examples of this swift adaptive conduct include transitioning from working at corporate headquarters and studios to working remotely from home, navigating the range of government financial assistance and employee retention programmes such as JobKeeper (Australian Tax Office, 2020), and closing retail stores in order to comply with state government lockdowns (interviews).

At the same time, our participants reported working to mitigate the impacts on their businesses of supply chain disruptions such as delays with fabric suppliers, the closure of off‐shore production facilities, increased freight costs, cancelled orders, and more (interviews). As evidenced in the next section, experiences in this regard varied according to location(s) of production facilities.

Interviews also highlighted the extremely high levels of stress individuals have felt during the pandemic, with many describing a sense of deep personal responsibility for the health and wellbeing and economic security of their staff. As one participant told us:

I'm exhausted. First, there was the initial panic of the unknown, which was mainly health related. Then, there was the business. There are probably a hundred people that rely on their salaries from my company. I felt responsible for all of them. I'm also trying my best to keep an eye on my own mental health and wellbeing. The fact that the kids are back at school has been a big help too. I'm praying we don't go into lockdown part two … (Brand D, head designer and owner)

Yet, despite the fact that brand representatives and owners and their teams are working in isolation and focused on mitigating the immediate financial impact of the pandemic on their individual businesses, we found that they were also highly collaborative during 2020. In particular, the AFC was highly active in supporting initiatives to foster collaboration, such as virtual roundtables (initiated by Showroom X), and webinars to help members of the industry navigate this uncertain time (AFC, 2020b). As one participant told us, “at the start, there were 20 or 30 of us but over time it grew to around 200 brands. It was really nice to see the industry coming together, talking about the issues, how we can move forward and what we can do to support each other” (Brand A, co‐founder).

Additional examples of industry collaboration on business‐to‐consumer initiatives included the shop‐local campaign #wewearaustralian, organised by a group of Australian fashion designers including Bassike, Carla Zampatti, Nobody Denim, R.M. Williams, and the Upside, which offered discounts on new‐season merchandise while also making donations to local charities^7^ (McIlvaine, 2020a), and providing discounts during Vogue Australia's 48‐hour ‘Fashion Relief’ campaign (Vogue Australia, 2020). Department store David Jones launched a ‘Home of Australian Fashion’ campaign to further support local designers (David Jones, 2020).

Moreover, in light of the cancellation of Australian fashion week, with ORDRE, the AFC created a virtual business‐to‐business showroom to help Australian fashion brands share their collections with international buyers (Press, 2020). Given the growing importance of international markets for Australian fashion brands, and in light of ongoing lockdowns and limited international travel for fashion weeks, virtual showrooms may become crucial for brands to connect with fashion buyers.

Taken together, such evidence suggests that despite isolation enforced by the pandemic, which significantly altered the geographies of work for fashion brands and their employees, there were still high levels of collaboration and mutual support within the industry during its early stages.^8^

### 4.2 Impact on garment production

The fashion industry relies upon highly globalised production networks, which were immediately disrupted by lockdowns and border closures related to COVID‐19 (Brydges & Hanlon, 2020). The pandemic created myriad challenges for Australian fashion brands with respect to production. Among them was the need to identify which garments could still be produced and delivered while incurring “emergency' surcharges” that resulted in increased freight and transportation costs and which orders would not be delivered. At the same time, industry players had to strategically pivot production, such as by moving away from tailored womenswear and introducing capsule collections of comfortable garments designed for working from home during the Australian winter (interviews).

Despite the decline of garment manufacturing capabilities in Australia, local garment production continues to exist. It is estimated that the sector currently employs approximately 37,000 people and produces 8% of the clothing produced in Australia (Sewport, 2019). Interviews revealed that many Australian fashion brands engage in a mix of highly specialised local and global production networks. For example, it is common for a brand to work with local garment production facilities to produce part of a collection and with international factories to compensate for a lack of domestic technical capabilities, as is the case for the production of knitwear.

During the pandemic, Australian fashion brands working with local manufacturers found themselves at a competitive advantage. For example, during an interview, the Operations Manager for Brand B described being able to move a new collection consisting of ‘comfy knitwear and Zoom tops’ aimed at consumers working from home from design to retail in just four weeks. This result supported domestic employment across the industry and injected much needed cash‐flow into the business. It illustrates how local production can be attuned to changing market conditions and consumer demands. By comparison, participants working with international manufacturers described experiencing either good or bad “luck” depending on whether their factories were affected by COVID‐related border and/or facility closures (interviews). This kind of experience is leading some designers to rethink their entire business model. As the founder of Brand H told us, she is taking time to “reset” after her workshop in India was closed because of COVID‐19 cases and subsequent lockdowns. It is presently unclear what form her new brand may take.

Sector agility was also evident in how members of the Australian fashion industry pivoted to produce personal protective equipment, among them Scanlan Theordore (Ragtrader, 2020) and Ethical Clothing Australia accredited brands such as Nobody Denim, Cue, and The Social Studio (Ethical Clothing Australia, 2020). Canberra‐based sustainable fashion label Pure Pod also engaged in a campaign for the public to “Adopt a Medical Professional” by creating scrubs—addressing an urgent need in the community while simultaneously supporting the business (Crowe, 2020).

Thus, despite significant challenges, members of a highly skilled garment manufacturing sector have been redefining the Australian fashion industry over time and helping to make segments of the industry more resilient in the face of crisis.

### 4.3 Impact on fashion retail

As designer Camilla Franks told*The Daily Telegraph*, from new safety procedures to a groundswell of support for local brands, fashion retail will be forever changed by COVID‐19 (Nsenduluka, 2020). Doubtless, Australian fashion brands have been highly innovative in responding to lockdown‐mandated store closures by harnessing the opportunities of online retailing. For example, the AFC found that over 50% of respondents to its survey introduced new digital marketing and sales programmes, which was a finding confirmed during our own interviews.

Digital retailing strategies identified during those interviews include improvements to and investments in the online shopping experience such as live chats with sales associates and more detailed product descriptions, virtual sample and/or warehouse sales and other promotions to support immediate cash flow objectives, and the use of Instagram to sell, promote, and connect with consumers. Social media is particularly important. As the co‐founder of Brand D told us, “Of course, we have a website but Instagram is entirely different. You can market and sell your products and become a global brand in a way that 10 years ago would not have necessarily been possible.”

Whereas brands with a strong online presence before the pandemic have been particularly successful in using online platforms to bolster their businesses and have recouped a significant percentage of lost sales,^9^ those without such platforms have had to “play catch up” and rapidly develop their online presence in order to attempt to access consumers (interviews). As the operations manager for Brand B told us, “in light of COVID, we've woken up to the importance of online sales. Luckily, two months before COVID we made the decision to hire a full‐time online manager and are now making even further investments into our digital presence we otherwise wouldn’t have made.”

Given the inroads made with online retailing and uncertainties regarding if and when physical retailing will return to “normal”, particularly in light of new lockdowns such as that in Melbourne in August 2020 (cf. McIlvaine, 2020b), our participants described having doubts about the need for, and about the status of future investments in, multiple retail locations. In Australia, outdoor retail spaces are also predicted to make gains at the expense of indoor malls (Williams, 2020). This shift may have significant implications for the future geographies of retail. Before COVID, Australia was home to some of the highest commercial rents in the world (Stilinovic, 2018), which was already a disincentive for fashion brands to open physical stores. Thus, for example, it may be that over time brand leads will decide to maintain “flagship” retail locations while closing secondary stores, which would have considerable implications for high streets, malls, and neighbourhoods and individuals and communities in those locales.

Finally, although before COVID it was estimated that the average Australian spent nearly $100 a month on clothing and shoes (Craik, 2015), and despite ongoing economic uncertainty, it seems as though spending on fashion has already begun to recover because some consumers are shopping once again (interviews). This change has helped bolster the recovery of Australian fashion brands thus far. Yet there are also indications that consumer spending patterns are beginning to change:

The economic hardship felt by many will mean customers won't be making purchasing decisions lightly—they'll be more discerning in what they buy and why, likely focusing on cost‐per‐wear and garment longevity. We also anticipate that this time spent at home will give customers the space to reflect on what they actually need in life, as well as what world they want to live in. We're seeing a strengthened focus on supporting local and in customers wanting to align with brands that reflect their personal values. (Intermediary G, corporate sustainability officer)

These changes in focus and aligment may ultimately be to the advantage of Australian brands with messages related to local, sustainable, and ethical production.

## 5 DISCUSSION AND CONCLUSION

Now that we have traced the evolution of Australia's domestic fashion industry and the impact of COVID‐19 on the geographies of fashion across spaces of design, production, and retail, we can consider the implications for future pathways of the industry. As our findings reveal, using a path‐dependency framework has allowed us to contextualise the shock of COVID‐19 against the historical evolution of the industry and better understand both the opportunities and challenges facing the industry today.

By taking an evolutionary approach, we have identified several existing, historical industrial strengths that have supported the Australian fashion industry's response to COVID‐19 thus far. These strengths include the knowing and valuing the importance of (a) highly organised industry associations able to encourage collaboration, facilitate knowledge transfer, and advocate for the sector to the public and policymakers during a highly uncertain time, and (b) local garment manufacturing facilities and their role in supporting brands' abilities to continue to make clothes (both scheduled collections and new capsules attuned to changing consumer demands) with relatively few disruptions.

Many fashion design brands have been experiencing a rise in popularity both domestically and abroad in recent years, and many have also been innovative and adaptable in their responses to the significant, immediate impact of COVID‐19 on their businesses, whether that has involved finding new ways to connect with consumers and buyers both locally and abroad, investments in digital retailing and promotions, or keeping staff employed through JobKeeper. There is also evidence of Australian fashion designers creating new pathways by expanding into homewares, a sector that has experienced renewed popularity during the pandemic (Rocca, 2020).

COVID‐19 has also reinforced the importance of having strong brand aesthetics and identities that resonate with consumers. Here, there is a particular opportunity for brands with a sustainability mandate (including those tied to domestic garment production) to tap into changing consumer sentiments and reflect on their consumption patterns. That opportunity is building momentum around value‐driven brands. We have also highlighted the emotional labour that such work entails, which has only increased during this time (Heinze, 2020).

Building on work by Rantisi (2004), we have found examples of where the COVID‐19 pandemic has led to the acceleration of industrial disruption and the emergence of new pathways in the industry. The ongoing impact of COVID‐19 may challenge the historical dominance of Sydney and Melbourne in Australian fashion design. If working from home becomes further entrenched as the “new normal” and if collaboration increasingly takes place online there may be new opportunities for designers who were already operating in secondary fashion cities such as Canberra, Brisbane, or Perth to regain relevance (Tuite, 2019b) and be further engaged in industry conversations without the added burden of travel or relocation to be physically present in core cities (see also Brydges & Hracs, 2019). The prospect that one can build an international brand from within Australia may help to reduce concerns about the brain drain of domestic design talent to leading fashion capitals offshore. However, regardless of domestic home base, brands will need to find ways to access international markets without traditional avenues such as fashion weeks for the foreseeable future.

In addition, although there are concerns that the pandemic will stymie investments in industry sustainability initiatives (Brydges et al., 2020), our research has found that over 2020 some brands were actually increasing investments in such initiatives. As the corporate sustainability officer for a multibrand retailer (Intermediary G) told us:

Initially, we had to press pause on certain sustainability initiatives as we were forced to focus on the direct, uncertain impacts of COVID‐19. As time has passed, it has reinforced just how important sustainability is to our values. Conversations we were having pre‐COVID have been accelerated and we are strengthening our commitment to support initiatives with positive social, environmental and community impacts.

Thus, at this early stage in the pandemic landscape, we see some signs that alongside local production, sustainability may be a growing priority in the Australian fashion industry and could become another source of competitive advantage. And, as the industry continues to look for solutions, there are opportunities to learn from and collaborate more with Indigenous designers (Campbell, 2020).

And finally, although local manufacturing has been an invaluable asset during the pandemic, a dominant theme arising from our interviews was that investment is needed to bolster the industry, particularly to train new workers to replace an ageing labour force and purchase new machinery (see also Craik, 2015). Additional insights to emerge from this research relate to the need for public procurement policies that would require domestic production to encourage capital investment in the sector, targeted financial support for brands that produce locally, educational support for fashion production, and business‐to‐business resources to connect designers looking to manufacture locally with domestic manufacturing facilities.^10^ This last point is also an instance of where locally oriented policy (cf. Couch et al., 2011) could strengthen this segment of the fashion industry.

Although our research offers a snapshot in time, we have shown that a path‐dependency approach enables us to place the impact of COVID‐19 on the Australian fashion industry in context. As this situation continues to unfold, future research will be needed to continue to investigate the impact of COVID‐19 on the Australian economy more broadly and the fashion industry in particular. Specifically, more research will be needed to understand the challenges and opportunities facing the garment manufacturing sector, which may also have important policy implications.

## ENDNOTES

1 Although difficult to find precise numbers, it has been estimated the Australian fashion industry was worth AUD$28.5 billion in 2016 (Fashion United, 2016).

2 See Peers (2009) for an overview of Australian fashion before 1945, which is beyond the scope of this paper.

3 See Tuite (2018) for an in‐depth examination of work by Jenny Kee and her Flamingo Park retail store, and the important role each played in the evolution of the Australian fashion industry during the 1970s.

4 As the decade progressed, online retailers such as Asos and The Iconic also gained considerable traction, accounting for 10% of total retail spending (BBC, 2019). One result was that, between 2017 and 2019, several well‐known Australian fashion brands entered voluntary administration, including David Lawrence, Rhodes and Beckett, and Maggie T (BBC News, 2019).

5 For example, the popular American style blog “Who What Wear” recently ran an article reflecting both these trends, describing Australian fashion in the following terms: “we often associate Australia with sun, fun, and everything they entail, but what we frequently fail to realize is just how objectively fashionable it secretly is” (Eggertsen, 2020).

6 The survey featured 182 respondents from a range of sectors (including retail (online/offline), wholesale design, and designer labels) and scale (including micro, small, medium, and large/international businesses with revenue ranging from less than AUD$1 million annual turnover to more than AUD$50 million).

7 Campaign co‐founder, Richard Poulson, told *Inside Retail*: “We are all in this together, so my belief is that we all have the same issues, whether it be business, family or personal and being able to share experiences with each other will help each of us to get through this crisis. We need to look after each other” (Poulson quoted in McIlvaine, 2020a, n.p.)

8 However, we also found that participants, particularly those from smaller brands, experienced less engagement in some or all industry initiatives. For some, this level of engagement reflected the need to focus inward on myriad, competing tasks their own brand demanded, whereas others described feeling disconnected from industry intermediaries even pre‐COVID and were hesitant to commit to new engagements at this time.

9 It was reported that fashion retail sales were down 22% in June 2020, which, although not ideal, is an improvement over the retail sales figures from the beginning of the pandemic (ABC News, 2020).

10 The role of the Australian government was often tied to these discussions, and as a media release from the Minister for Industry, Science and Technology, Karen Andrews MP, detailed, there may be interest in bolstering domestic production capabilities: “Now more than ever it's important we all support Australian‐made products where we can because the flow‐on effects help keep people in jobs, allow small businesses to become part of previously off‐shore supply chains, and open the door for new export markets in the long term” (Andrews, 2020).

## Appendix A LIST OF INTERVIEW PARTICIPANTS

**Table:**

Participant	Role	Industry segment	Some or all production in Australia	Location	Gender
Fashion brands					
Brand A	Brand co‐founder	Designer menswear and womenswear, casual	Yes—majority	Sydney, NSW	Female
Brand B	Brand operations	Designer workwear for women	Yes—majority	Sydney, NSW	Female
Brand C	Brand co‐founder	Designer workwear and evening apparel	Yes—some	Brisbane, QLD	Female
Brand D	Head designer and owner	Womenswear	Yes—all	Sydney, NSW	Female
Brand E	Brand co‐founder	Luxury resortwear	No	Sydney, NSW	Female
Brand F	Brand co‐founder	Contemporary womenswear	Yes—all	Sydney, NSW	Female
Brand G	Brand co‐founder	Designer womenswear, bathing apparel	Yes—majority	Brisbane, Sydney	Female
Brand H	Brand founder	Ethical womenswear and children's clothing	No	Sydney, NSW	Female
Brand I	Brand operations	Womenswear and menswear, including denim	No	Brisbane, QLD	Female
Brand J	Brand founder	Womenswear, maternity wear	All	South Coast, NSW	Female
Brand K	Brand founder	Womenswear	All	Sydney, NSW	Female
Other industry actors					
Intermediary A	Senior management	Industry association	N/A	Melbourne, VIC	Female
Intermediary B	Senior management	Industry association	N/A	Melbourne, VIC	Female
Intermediary C	Senior management	Social enterprise	N/A	Sydney, NSW	Female
Intermediary D	Founder	Retail (pop‐up + online)	N/A	Sydney, NSW	Female
Intermediary E	Corporate sustainability officer	Retail (online, direct‐to‐consumer)	N/A	Sydney, NSW	Female
Intermediary F	Founder	Retail (rental)	N/A	Canberra, ACT	Female
Intermediary G	Corporate sustainability officer	Retail (in‐store and online multibrand retailer)	N/A	Melbourne, VIC	Female
Intermediary H	Founder and store manager	Retail (second hand, in‐store and online)	N/A	Sydney, NSW (additional locations: Melbourne, VIC; Brisbane, QLD)	Female
Intermediary I	Founder	Retail (rental)	N/A	Sydney, NSW	Female
Intermediary J	Senior management	Retail (online + in‐store)	N/A	Sydney (+Brisbane and Melbourne)	Female
Consumers					
Consumer A	N/A	N/A	N/A	Sydney, NSW	Female
Consumer B	N/A	N/A	N/A	Sydney, NSW	Female
Consumer C	N/A	N/A	N/A	Sydney, NSW	Female
